# Multi-Target Effect of Aloeresin-A against Bacterial and Host Inflammatory Targets Benefits Contact Lens-Related Keratitis: A Multi-Omics and Quantum Chemical Investigation

**DOI:** 10.3390/molecules28196955

**Published:** 2023-10-06

**Authors:** Jency Roshni, Sheikh F. Ahmad, Abubakar Wani, Shiek S. S. J. Ahmed

**Affiliations:** 1 Drug Discovery and Multi-Omics Laboratory, Faculty of Allied Health Sciences, Chettinad Hospital and Research Institute, Chettinad Academy of Research and Education, Kelambakkam 603103, Tamil Nadu, India; 2 Department of Pharmacology and Toxicology, College of Pharmacy, King Saud University, Riyadh 11451, Saudi Arabia; 3 Department of Immunology, St. Jude Children’s Research Hospital Memphis, Memphis, TN 38105, USA

**Keywords:** keratitis, aloeresin-A, phytochemicals, docking, inflammation, cornea

## Abstract

Contact lens-mediated microbial keratitis caused by *Pseudomonas aeruginosa* and *Streptococcus pneumoniae* provokes corneal damage and vision loss. Recently, natural phytochemicals have become complementary medicines for corneal destruction. Herein, we aimed to identify multi-targeting *Aloe vera*-derived phytochemicals capable of inhibiting bacterial and host targets of keratitis through ADME (absorption, distribution, metabolism, and excretion), docking, molecular dynamics (MD) simulation, MMGBSA (molecular mechanics generalized Born surface area) and density functional theory (DFT) investigations. An extensive literature search revealed ExoU, ExoS, ExoT, ExoY, and PLY as virulent bacterial targets. Simultaneously, differential gene expression (DGE) and pathway enrichment analysis-specified host transcription factor (SPI1) influences keratitis pathogenesis. Molecular docking analysis uncovered aloeresin-A as a promising inhibitor against bacterial and host targets, demonstrating strong binding energies ranging from −7.59 to −6.20 kcal/mol. Further, MMGBSA and MD simulation analysis reflect higher binding free energies and stable interactions of aloeresin-A with the targets. In addition, DFT studies reveal the chemical reactiveness of aloeresin-A through quantum chemical calculations. Hence, our findings show aloeresin-A to be a promising candidate for effectively inhibiting keratitis. However, additional research is imperative for potential integration into lens care solutions.

## 1. Introduction

Contact lenses are worn by over 150 million people worldwide to correct their refractive impairments [1]. Yet, extensive use of these lenses provides a wide range of complications, from superficial punctate keratitis to potentially blinding infectious keratitis [2]. The major cause of infectious keratitis is poor hygiene and contaminated contact lens cases [3]. Clinical manifestations include pain, mucopurulent discharge, photophobia, and redness. About 90% of infectious keratitis is caused by bacterial species [4]. Bacterial keratitis is primarily characterized by corneal ulcers or infiltrates, stromal loss, and overlying epithelial defect [5]. *Pseudomonas aeruginosa*, *Streptococcus pneumoniae*, and *Staphylococcus aureus* contribute over 80% of bacterial corneal ulcers by adhering to the base of an epithelial defect. Notably, *P. aeruginosa*, the most frequent ocular pathogen, perforates the cornea within 72 h using their virulence effector proteins [6]. They utilize a type III secretion system to deliver these effector proteins into host cells [7]. In particular, ExoU, secreted exoenzyme S (ExoS), exoenzyme T (ExoT), and adenylate cyclase (ExoY) are the four effector proteins (exotoxins) that have been examined so far in *P. aeruginosa*. ExoU causes rapid lysis of host cells through its potent phospholipase activity. Both ExoS and ExoT possess GTPase activating protein (GAP) and ADP ribosyltransferase (ADPRT) activities that impair cytoskeletal function, cell migration, phagocytosis, and epithelial cell barriers in host cells. Contrarily, ExoY was found to disrupt the actin cytoskeleton [8]. In the case of *S. pneumoniae*, pneumolysin (PLY), a member of cholesterol-dependent cytolysins, promotes host cell lysis in the keratitis condition [9]. Moreover, *P. aeruginosa* and *S. pneumoniae* exotoxins were also found to activate the host immunological pathways by producing several inflammatory mediators [10,11]. These inflammatory mediators continuously recruit polymorphonuclear leukocytes to the site of infection, ultimately causing corneal tissue destruction [10].

Contact lens care solutions have been developed to minimize bacterial growth or contamination on lenses [12]. Nevertheless, these solutions compromise the integrity of the corneal epithelium and cause discomfort [13]. Natural compounds are safe on the eyes and can kill infectious pathogens [14]. *Aloe vera*, a drought-resistant perennial plant, possesses multiple biological roles, such as antioxidant, anti-inflammatory, antibacterial, antifungal, and antiviral properties [15]. For instance, an experimental study on the colitis model highlights the anti-inflammatory effects of *A. vera* through various molecular and histological parameters [16]. *A. vera* also shows potential ameliorating effects in oxidative stress-related diabetes and bacterial and fungal conditions [17,18,19]. Recently, *A. vera*-based eye drops were investigated in treating oxidative stress-mediated corneal deterioration [20]. Such potential therapeutic values of *A. vera* were attributed to its bioactive components [21]. Therefore, finding the potential bioactive compounds of *A. vera* against *P. aeruginosa* and *S. pneumoniae* and incorporating them in a lens care solution could protect the ocular surface epithelium from keratitis.

**Figure 1:**
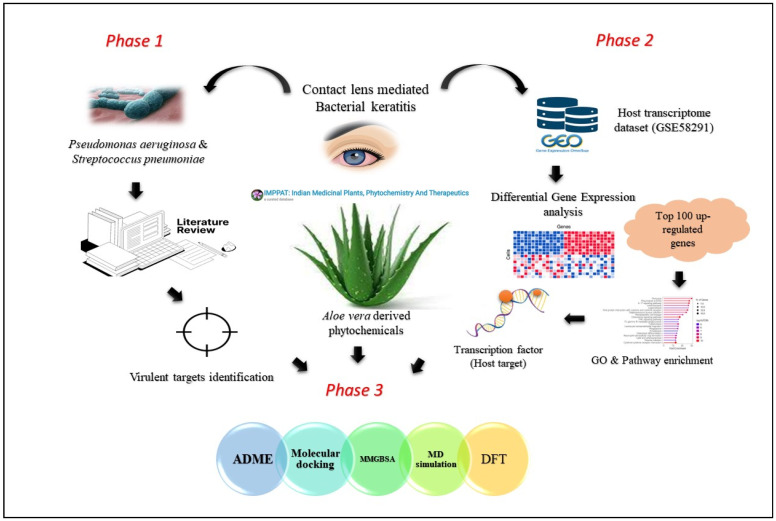
Computational workflow involving identification of bacterial target proteins in contact lens-mediated keratitis (Phase 1); differential gene expression (DGE) and pathway enrichment analysis-specified host transcription factor (SPI1) influencing keratitis pathogenesis (Phase 2); screening phytochemicals from *Aloe vera* for their inhibitory effects on these targets using various computational methods, including ADME, molecular docking, molecular dynamics simulation, MMGBSA, and density functional theory (DFT) (Phase 3).

This study aimed to identify potential *Aloe vera*-derived phytochemicals capable of inhibiting bacterial targets and the host regulators of keratitis. An extensive literature search was performed to identify the bacterial targets. We also analyzed keratitis-related transcriptome data to pinpoint upregulated genes to discover the host target influencing keratitis. Additionally, through KEGG pathway enrichment analysis, we identified the inflammatory signaling pathways of these upregulated genes. Further, the key transcriptional factor (TF) regulating the crucial upregulated genes and influencing the keratitis pathogenesis was examined. Finally, we screened phytochemicals of *A. vera* for their inhibitory effect against these targets by implanting a series of computational methods such as absorption, distribution, metabolism, and excretion (ADME), molecular docking, MMGBSA (molecular mechanics generalized Born surface area), molecular dynamic (MD) simulation, and density functional theory (DFT) (Figure 1). Our investigation showed aloeresin-A as a potent inhibitor against the bacterial and host targets with significant binding affinity. MMGBSA and MD simulation studies further revealed the interaction strength and stability of aloeresin-A with bacterial and host targets. Further, the DFT method identifies aloeresin-A’s structural, chemical, and electronic properties, thus aiding in comprehending its ability to interact with target proteins.

## 2. Results and Discussion

Microbial keratitis is considered to be the most common corneal infection related to contact lens wear [22]. Gram-positive and Gram-negative bacterial species contribute to the pathogenesis of keratitis [6]. An increasing body of evidence reports the pathological association of exotoxins (ExoU, ExoS, ExoT, and ExoY) of *P. aeruginosa* and pneumolysin (PLY) of *S. pneumoniae* in bacterial keratitis [7,10,22]. In addition to the bacterial causative factors, increased levels of proinflammatory factors and neutrophils at the site of infection worsen the corneal tissues [23]. Hence, developing contact lens care solutions by targeting both bacterial and host targets is vital to prevent contact lens-related keratitis further.

### 2.1. Literature Analysis and Bacterial Target Identification

Using the target selection criteria described in the methodology section, the highly keratitis-reported exotoxins of *P. aeruginosa*, such as ExoU, ExoS, ExoT, and ExoY, were collected. Similarly, for *S. pneumoniae*, pneumolysin (PLY) protein was selected as a target protein. With no availability of experimental structures in the PDB database, the appropriate predicted 3D structures of bacterial targets, ExoU (A0A221LFV5), ExoS (G3XDA1), ExoT (Q9I788), ExoY (A0A221LFV5), and PLY (Q7ZAK5) were collected from the Alpha-Fold structural database using their accession IDs.

### 2.2. ADME Profiling and Ligand Collection

A list of 56 *A. vera*-derived phytochemicals and their structures was downloaded from the IMPPAT (Indian Medicinal Plants, Phytochemistry and Therapeutics) database in spatial data file (SDF) format [24]. The major characteristics of these phytochemicals were terpenoids, polyketides, alkaloids, and carbohydrates. Among 56 collected phytochemicals, 48 were found druggable by their ADME compliance scores (star values (0–5): high-druggable properties) using the QikProp module of Maestro-Schrödinger version 11.2 [25]. After ADME screening, 48 phytochemicals with drug-likeness properties were exported for docking against ExoU, ExoS, ExoT, ExoY, and PLY bacterial targets. In contrast to previous research by Goudarzi et al. [15] and Arbab et al. [26], who focused on the effectiveness of *A. vera* crude extract against several bacterial species, our study appears to be the first to examine the antibacterial properties of its bioactive phytochemicals specifically. Hence, as evidenced above, the ADME-filtered *A. vera* (*n* = 48) phytochemicals were exported for molecular docking to investigate their binding affinities with the bacterial targets.

### 2.3. Regulatory Network Construction and Host Target Discovery

**Figure 2:**
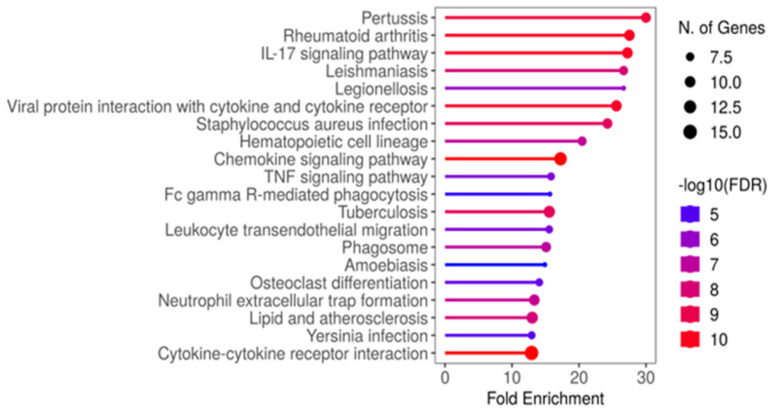
Molecular pathway enrichment analysis for top 100 upregulated genes. Enriched pathways are ranked by fold enrichment values. The most significant pathways are highlighted in red, and the less significant pathways are highlighted in blue according to log10(FDR) values. Larger dots in the graph represent a higher number of genes involved.

**Figure 3:**
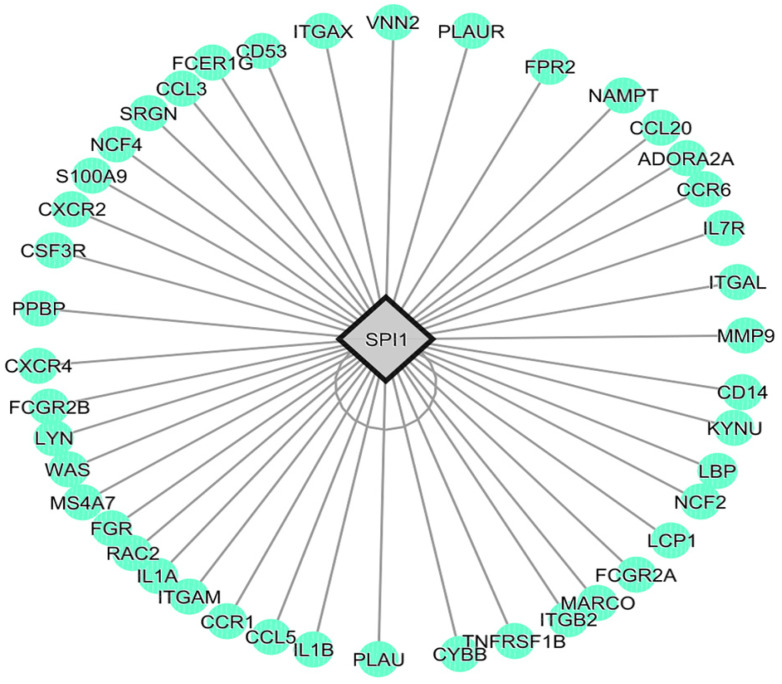
Transcription factor analysis showed SPI1 could be the potential host target regulating 43 genes encoding proteins associated with contact lens-mediated keratitis. Gray node represents transcription factor, green nodes represent targets. Gray lines represent connectivity.

A suitable transcriptomic dataset (Accession ID GSE147507) was collected to identify potential keratitis-related host targets. The dataset included 27 tissue samples (seven bacteria-infected corneas, eight fungi-infected, and 12 healthy corneas). Only bacteria-infected cases, along with their respective controls, were chosen for our DGE analysis. The cut-off criteria were set as FDR < 0.05, |log 2FC| > 0. 1, and *p*-value < 0.05 to assess the differential expressed genes (DEGs). A total of 11607 DEGs (3711 up and 4584 downregulated) were identified by comparing the bacteria-infected and healthy corneal tissues. Of 3711 upregulated genes, the significant top 100 upregulated genes (Table S1) were taken for pathway enrichment analysis. Using the 100 upregulated genes with ShinyGO [27], the most significantly enriched inflammatory pathways were IL-17 signaling, chemokine signaling, and tumor necrosis factor (TNF) signaling (Figure 2). Hence, the top 100 upregulated genes in keratitis were found to be highly associated with inflammatory conditions. Accordingly, the TF regulating most of the top 100 upregulated genes underlying inflammation was identified by the iRegulon plug-in, Cytoscape [28]. The transcription factor SPI1 was found to regulate 43 genes encoding protein in the list (Figure 3) and considered as the potential host target. Interestingly, Han et al. [29] and Xia et al. [30] suggest the SPI1 transcription factor as a potential target to mitigate inflammation. Accordingly, inhibiting host SPI1 prevents binding to the DNA and might modulate the expression of its regulating genes associated with the inflammatory processes. Hence, the 3D structure of host target SPI1 (Accession ID: P17947) was retrieved from the Alpha-Fold database for the subsequent molecular docking studies.

### 2.4. Inhibitory Potential of A. vera Phytochemicals against Keratitis Targets

**Figure 4:**
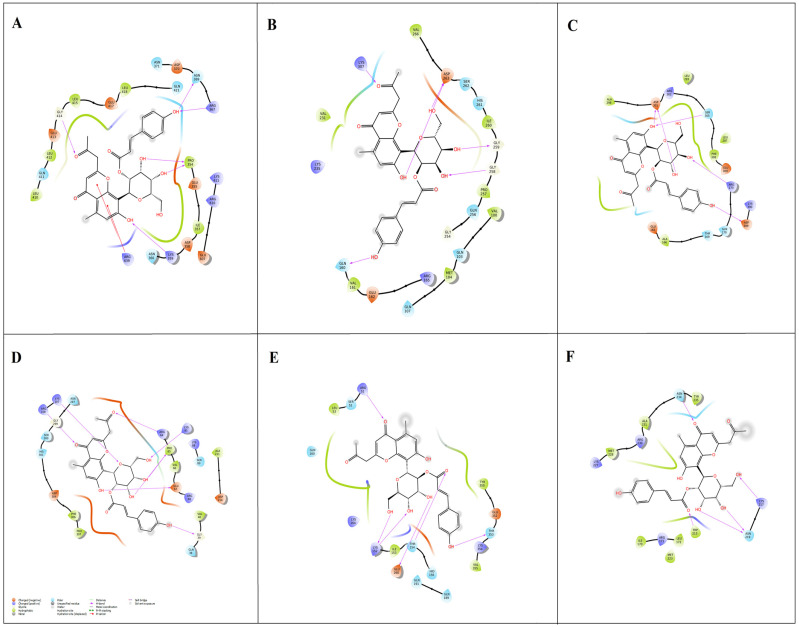
Two-dimensional interaction plots of aloeresin-A with ExoU (**A**), ExoS (**B**), ExoT (**C**), ExoY (**D**), PLY (**E**), and SPI1 (**F**).

The *A. vera*-derived phytochemicals (*n* = 48) were docked with the bacterial (ExoU, ExoS, ExoT, ExoY, and PLY) targets to investigate their binding efficiencies. Several binding modes were predicted for the phytochemicals across the targets. A list of top-five phytochemicals showing high binding affinity to each target was chosen (Table 1). Among the docked phytochemicals, aloeresin-A exhibited a high binding affinity towards all the bacterial targets (ExoU, ExoS, ExoT, ExoY, and PLY) in the range of −7.59 to −6.20 kcal/mol. The docked aloeresin-A with ExoU achieved a binding score of −7.59 kcal/mol with six hydrogen bonds at GLY414, ASN369, ARG367, PRO354, LYS359, and ARG639 residues (Figure 4A). With regard to ExoS, the aloeresin-A formed six hydrogen bonds at ASP263, GLY259, GLY258, LYS309, and GLN160 residues with −7.09 kcal/mol binding energy (Figure 4B). With ExoT, aloeresin-A formed five hydrogen bonds at ASP301, SER305, ARG173, and ASP389 residues (−6.56 kcal/mol) (Figure 4C). Seven hydrogen bonds were formed between aloeresin-A and ExoY (−6.42 kcal/mol) at ARG108, LYS107, ARG64, LYS81, PRO65, GLU67, and GLY39 residues (Figure 4D). Aloeresin-A formed six hydrogen bonds with PLY protein at ARG51, LYS152, THR154, GLU260, and THR353 residues, resulting in an energy value of −6.20 kcal/mol (Figure 4E). Hence, aloeresin-A was further examined for its binding efficiencies against the host SPI1 target. With regard to host target SPI1 (Figure 4F), aloeresin-A formed five hydrogen bonds at ASN234, LYS217, ASN219, and TRP213 residues with a binding score of −5.62 kcal/mol. Interestingly, in this last case, aloeresin-A showed the least binding energy (of −5.62 kcal/mol). Earlier, Breaud et al. [31] performed LC-MS profiling and discovered the antioxidant effects of aloeresin-A. Nevertheless, our study appears to be the first to investigate the antibacterial and anti-inflammatory effects of aloeresin-A in silico.

### 2.5. Binding Free Energy Calculations

The multi-target binding aloeresin-A and polyquaternium were further selected to compute binding free energies using the MMGBSA approach [32]. The MMGBSA calculations were performed to estimate the relative binding affinity between target proteins and ligands. The calculated free energy (∆Gbind) values (Table 2) of ExoU-aloeresin-A, ExoS-aloeresin-A, ExoT-aloeresin-A, ExoY-aloeresin-A, PLY-aloeresin-A, and SPI1-aloeresin-A were −57.36, −54.89, −56.98, −50.72, −59.18, and −49.21 kcal/mol, respectively. Our findings show that aloeresin-A showed lower binding free energy values, which is also supported by our docking scores. Therefore, MD simulation was performed to assess the conformational stability of target proteins with the best binding aloeresin-A.

### 2.6. Dynamic Simulation of Aloeresin-A against Bacterial and Host Targets

**Figure 5:**
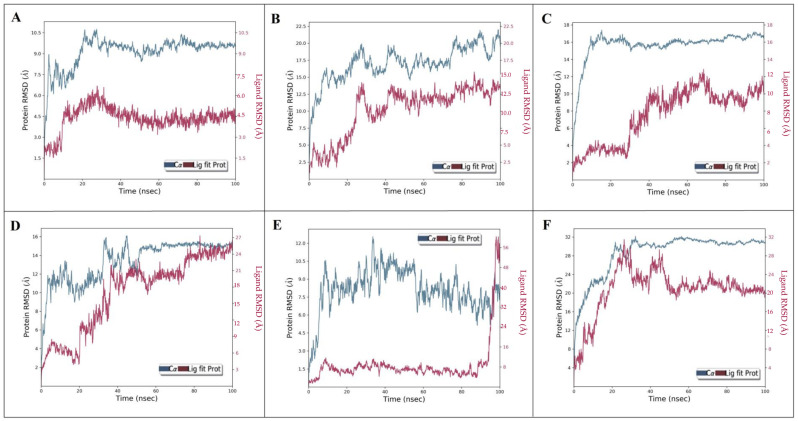
RMSD (C-alpha) analysis of aloeresin-A with ExoU (**A**), ExoS (**B**), ExoT (**C**), ExoY (**D**), PLY (**E**), and SPI1 (**F**). The red color denotes Lig fit on protein, and the blue represents protein RMSD.

**Figure 6:**
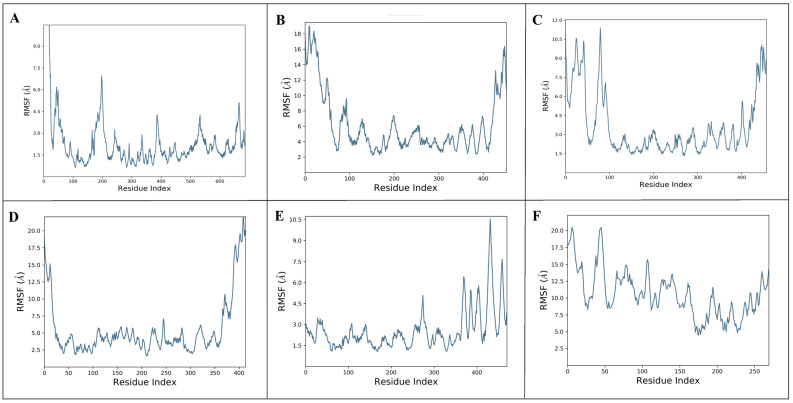
Protein RMSF analysis of aloeresin-A with ExoU (**A**), ExoS (**B**), ExoT (**C**), ExoY (**D**), PLY (**E**), and SPI1 (**F**).

The multi-target behavirs of aloeresin-A were further assessed for their stability with targets (ExoU, ExoS, ExoT, ExoY, PLY, and SPI1) using Desmond MD simulation [33]. The stability of protein–ligand complexes (ExoU-aloeresin-A, ExoS-aloeresin-A, ExoT-aloeresin-A, ExoY-aloeresin-A, PLY-aloeresin-A, and SPI1-aloeresin-A) were analyzed based on the root-mean-square deviations (RMSD), root-mean-square fluctuations (RMSF) trajectories. The dynamic behavior of each complex was investigated through RMSD plots along with its calculated average values which generally measure the scalar distance between protein (Cα backbone) and ligand (Figure 5A–F). Upon binding of aloeresin-A, the RMSDs of ExoU (Figure 5A), ExoT (Figure 5C), and SPI1 (Figure 5F) were found to increase initially, reaching maximum values within the range of 10–32 Å till 40 ns, and later remained stable. In the case of ExoS (Figure 5B), the maximum deviation was observed at 22 Å and maintained stable equilibrium, whereas ExoY and PLY with aloeresin-A (Figure 5D,E) could not reach stable equilibrium till 100ns. However, aloeresin-A showed good binding affinity in both docking and MMGBSA analysis. Next, the flexibility of six proteins after aloeresin-A binding was assessed by calculating the RMSF of individual amino acid residues. The maximum fluctuations were observed at LEU198, SER11, ALA80, ALA410, ALA432, and MET7 for ExoU, ExoS, ExoT, ExoY, PLY, and SPI1, respectively (Figure 6A–F). In addition, notable fluctuations were observed at the binding site of aloeresin-A for each protein, thus confirming flexible binding (Figure 6A–F). Similarly, the ligand RMSF depicts the fluctuations of aloeresin-A atom by the atom when bound to the amino acid residues of six protein targets (Figure S1A–F). Subsequently, the protein–ligand plots (Figure S2A–F) explain the interactions during the simulation in and around the active site region through the formation of hydrogen bonds, ionic interaction, hydrophobic interaction, and water bridges. Furthermore, the timeline of protein–ligand interactions was plotted throughout 100 ns (Figure S3A–F). Overall, aloeresin-A interacted well with all analyzed targets of both bacteria and host, with a minimum of four contacts throughout the simulation period. Figure S3A–F also demonstrates the influence of secondary structure elements (SSE) on the overall stability of each complex.

### 2.7. Density Functional Theory Analysis

Aloeresin-A was further assessed for its structural, chemical, and electronic behavior using DFT to interact with target proteins. DFT also provides insights into the electron density distribution of small molecules, which are crucial aspects of protein binding [34]. All the quantum computational calculations (Figure S4A–D) were executed at B3LYP-D3/6-311**G level of theory in the Jaguar module of Schrödinger [35]. The molecular structure of aloeresin-A presented in Figure S4A contains 11 oxygen as heteroatoms. Calculated structural parameters such as bond lengths (Å), bond angles (°), and dihedral angles (°) were listed in Table S2. The bond length representing the distance between nuclei of two atoms was noticed to be greater for C12-C14 atoms with a value of 1.57 Å. The shortest bond distances were observed between O11-H67, O3-H47, O4-H48, O6-H50, and O7-H55 with a length of 0.96 Å. The shortest bond angle was seen at H42-C14-C17 with an angle of 101.15 Å. The largest bond angle was identified as 126.07 Å at C33-C32-C30. All these bond parameters influence the ligand’s ability to bind with proteins by affecting its shape, hydrogen bonding potential, and steric compatibility [36]. Therefore, the theoretically calculated bond parameters describe better shape and conformation of aloeresin-A regarding its binding ability with the target proteins.

#### 2.7.1. HOMO and LUMO Analysis

The frontier molecular orbitals include both the highest occupied molecular orbital (HOMO) and lowest unoccupied molecular orbital (LUMO) [34]. HOMO and LUMO values help to predict electron accepting/donating potential and significant reactive regions of chemical compounds. The HOMO (electron-rich orbital) value for aloeresin-A was −6.07 eV, denoting its ability to transfer electrons to unoccupied orbitals. The LUMO (orbital lacking electrons) value was found to be −1.70 eV for the phytochemical to accept electrons. HOMO and LUMO are primarily found on the right side of the molecule, revealing its susceptibility to charge transfer (Figure S4B) [37]. Additionally, Table 3 lists the various chemical descriptors of aloeresin-A. The energy gap between the highest and lowest molecular orbitals greatly influences the chemical reactivity and kinetic stability of molecules [38]. Aloeresin-A showed a smaller energy gap value of 4.37 eV, signifying better reactivity.

#### 2.7.2. Quantum Chemical Descriptors

Using HOMO and LUMO values, the quantum chemical descriptors were calculated with the help of mathematical equations presented in Table 3 [39,40]. High electron affinity (A) and ionization potential (I) indicate high electron acceptance and better chemical stability [39]. According to Table 3, aloeresin-A has good chemical stability with an electron affinity of 1.70 eV and an ionization potential of 6.07 eV. Aloeresin A was found to have a better capability of accepting (ω^+^ = 1.78 eV) and donating electrons (ω^−^ = 5.66 eV) [38]. The hardness of a molecule represents its resistance to changing electronic distribution, whereas softness indicates low resistance [38]. The calculated chemical hardness (η), global (S), and chemical softness (σ) were 2.18, 0.22, and 0.45 eV, respectively. The values of electrophilicity (ω = 3.45 eV), nucleophilicity (N = 3.11 eV), electronegativity (χ = 3.88 eV), and chemical potential (μ = −3.88 eV) describe the molecule’s electron donating and accepting abilities [38]. The lower chemical potential and higher electrophilicity index signify the electrophilic nature of aloeresin-A [40]. Thus, aloeresin-A was found to show high chemical reactivity with significant electronically stable features.

#### 2.7.3. Molecular Electrostatic Potential (MEP)

MEP displays electronic charge distribution in small molecules, aiding in predicting electrostatic interactions and binding affinities [38]. The MEP surface for aloeresin-A has been plotted in Figure S4C. The increasing order of electrostatic potential is red < white < blue. The positive electrostatic regions (blue) are associated with nucleophilic attacks, while negative electrostatic regions (red) are preferable for electrophilic attacks. These positive and negative electrostatic regions have been involved in bonded interactions throughout molecular docking and molecular dynamics simulations.

#### 2.7.4. Mulliken Charge Analysis

The charges on electrons greatly influence the molecule’s bonding capability [37]. The charge distribution of aloeresin-A is shown in Figure S4D and listed in Table S3. Notably, carbon 24 was found to have a higher positive value of 0.61 a.u. While carbon 34 had a lower value of −0.40 a.u. All the oxygen atoms were noticed to have negative charges, while the hydrogen atoms were positively charged. The negative region is associated with electrophilic reactivity, and the positive region behaves as a site for nucleophilic attack. Hence, the charge distribution of aloeresin-A deciphers both electron donors and acceptors, indicating more toward substitution reactions. Overall, a larger scale of electrophilicity was noticed for aloeresin-A in our findings, suggesting its chemical reactiveness. Consequently, this chemical reactivity implies that aloeresin-A could potentially engage in intermolecular interactions with a range of target proteins, offering promise for its use in treating keratitis, as suggested by our docking results. Although this study provides several benefits for multi-level phytochemical assessment, additional research is required to address the limitations of validation against the target.

## 3. Materials and Methods

### 3.1. Phytochemicals Collection and ADME Evaluation

*A. vera* phytochemicals were retrieved from the IMPPAT database (https://cb.imsc.res.in/imppat/, accessed on 12 February 2023) in spatial data file (SDF) format [24]. IMPPAT provides a variety of phytochemicals with relevant chemical and biological information. These phytochemical structures were optimized using LigPrep of Maestro-Schrödinger version 11.2 and inputted into the QikProp module of Maestro-Schrödinger version 11.2 [25] to filter based on their pharmacokinetic profiles. The phytochemicals, which displayed appropriate ADME compliance scores (indicated by stars value 0–5), were selected for further analysis.

### 3.2. Bacterial Target Search from the Literature

The two independent authors performed a systematic literature search to identify the reported targets of *P. aeruginosa* and *S. pneumoniae*. The literature search key terms include (1) contact lens-related keratitis, (2) drug targets for keratitis, (3) microbial target of keratitis, (4) exotoxins and keratitis, and 5) contact lens-bacterial toxins. Based on the gathered list of targets, the most influential bacterial target proteins were selected, and their corresponding 3D structures were searched in the PDB database (https://www.rcsb.org/search) using their name and official symbols. Due to the lack of experimental PDB structures, their predicted 3D structures were sourced from the Alpha-Fold database (https://alphafold.ebi.ac.uk), which provides an extensive collection of highly accurate predicted protein structures [41].

### 3.3. Host Target Identification through Regulatory Network Analysis

Next, the gene expression data relating to contact lenses and keratitis were searched in the NCBI GEO database. The relevant dataset describing the corneal transcriptomic profiling in bacterial and fungal keratitis (GSE58291) was identified. The GSE58291 dataset was based on the platform GPL10558 Illumina Human HT-12 V4.0 Expression BeadChip. From the GSE58291, we selected 12 healthy and seven bacteria-infected corneas for our analysis. Then, the limma R-program package was used to determine the differentially expressed genes between bacteria-infected and healthy corneal tissue with the cut-off criteria of |log 2FC| >0.1 and *p*-value < 0.05. To determine the underlying biological pathways of upregulated genes, KEGG enrichment analysis was performed using ShinyGo 0.77 (https://sdstate.edu) [27]. Then, the iRegulon plug-in [28] of Cytoscape 3.9.1 was used to identify the master transcription factor (TF) regulating the upregulated genes and designated as a host target for molecular docking analysis.

### 3.4. Molecular Docking with Bacterial and Host Targets

The target bacterial and host protein structures were prepared by adding hydrogen atoms, refining bond orders, creating disulfide bonds, deleting water molecules, and optimizing missing atoms using the PROPKA function of Protein Preparation Wizard, Maestro-Schrödinger version 11.2 [42]. The Glide module of Maestro-Schrödinger version 11.2 [43] was employed to perform molecular docking between phytochemicals against bacterial and host target proteins. Blind docking was executed to discover the likelihood of phytochemicals interacting with any region of the target proteins.

### 3.5. MMGBSA Analysis

The relative binding free energy (ΔGbind) for the selected phytochemical was calculated by incorporating Prime MM-GBSA, Maestro-Schrödinger version 11.2 [33], using the docked pose of the Glide algorithm. The ΔG bind formula is given below:

$$∆G\left(bind\right)=∆G\left(solv\right)+∆E\left(MM\right)+∆G(SA)$$

where ΔG(solv) signifies the difference in GBSA solvation energy of the protein–ligand complex and the total solvation energies of unbound protein and ligand; ΔE(MM) indicates the difference in the minimized energies between the complex and the sum of the unbound protein and ligand energies. ΔG(SA) reflects the difference in surface area energies of the protein–ligand complex and the sum I accept the correctionsof surface area energies for the unbound protein and ligand.

### 3.6. Molecular Dynamic Simulation

Following MMGBSA, the selected phytochemical that efficiently binds to both bacterial and host targets was taken for MD simulation. The dynamic effects of phytochemicals on the structures of bacterial and host proteins were analyzed via Desmond package, Maestro-Schrödinger version 11.2 [34]. Each protein–ligand complex was solvated through a single-point charge (SPC) water model with orthorhombic boundary conditions of size 10 Å. OPLS_2005 force field was employed for building the system, and charge neutralization was performed using Na^+^ and Cl^−^ ions. Next, the equilibrated simulation time was set to 100 ns with a recording trajectory of 4.8 PS, and the ensemble was consigned to NPT (300 K temperature and 1 bar pressure). Subsequently, the Simulation Interaction Diagram panel was used to analyze root-mean-square deviations (RMSD), root-mean-square fluctuations (RMSF), ligand–protein interaction plots, and the timeline of contacts between protein and ligand.

### 3.7. DFT Calculations

The quantum chemical calculations were carried out for best-bound phytochemicals using DFT. The DFT approach provides accurate electronic and structural information, enhancing the accuracy of binding predictions. All the calculations were performed at B3LYP-D3/6-311**G level of theory using the Jaguar module of Maestro-Schrödinger version 11.2 [35]. Structural parameters like bond angle, length, and dihedral angles were calculated theoretically. Various chemical descriptors such as frontier molecular orbitals (FMOs), energy gap (ΔE), ionization potential (I), electron affinity (A), electron accepting capacity (ω^+^), electron-donating capacity (ω^−^), chemical hardness (η), global softness (S), chemical softness (σ), electronic chemical potential (μ), electrophilicity index (ω), nucleophilicity index (N), and electronegativity (χ) were studied [40,41]. In addition, molecular electrostatic potential (MEP) and Mulliken charge distribution were computed.

## 4. Conclusions

Herein, we have demonstrated the potential application of aloeresin-A of *A. vera* as a component of lens care solutions to prevent keratitis via in silico evaluations. Our extensive literature search resulted in identifying virulent bacterial targets of keratitis. Differential gene expression and pathway enrichment analysis revealed the underlying pathological host transcriptional factor (SPI1) associated with contact lens-mediated inflammation. *A. vera*-derived phytochemicals that exhibited drug-likeness features through ADME profiling were exported for further studies. Our molecular docking, MMGBSA, and dynamic simulation showed the strong interaction of aloeresin-A with pathogenic bacteria and host targets. Additionally, DFT investigated the structural, chemical, and electronic characteristics that primarily contribute to intermolecular interactions for the multi-target binding aloeresin-A. Therefore, aloeresin-A may be a powerful inhibitor of keratitis targets, but extensive in vitro research is required for validation.

## Figures and Tables

**Table 1:** Docking scores of *Aloe vera*-derived phytochemicals.

SI.NO	Target	Phytochemicals	Docking Score (kcal/mol)
1	ExoU	Aloenin	−7.74
		**Aloeresin-A**	**−7.59**
		Isoaloesin	−7.38
		Aloin	−7.16
		Barbaloin	−7.16
2	ExoS	Aloin	−7.22
		Barbaloin	−7.22
		10-Hydroxyaloin B 6′-catate	−7.14
		**Aloeresin-A**	**−7.09**
		10-Hydroxyaloin A	−7.01
3	ExoT	**Aloeresin-A**	**−6.56**
		Isoaloesin	−6.35
		Aloenin	−6.07
		10-Hydroxyaloin A	−6.04
		7-Hydroxyaloin	−5.97
4	ExoY	**Aloeresin-A**	**−6.42**
		Aloenin	−6.20
		Aloin	−5.98
		Barbaloin	−5.98
		10-Hydroxyaloin B 6′-catate	−5.91
5	PLY	Allantoin	−6.89
		**Aloeresin-A**	**−6.20**
		Aloesin	−6.15
		Anthracene-1,8-diol	−6.12
		Isoaloesin	−5.90
6	SPI1	**Aloeresin-A**	**−5.62**

**Table 2:** MMGBSA calculations of aloeresin-A with target proteins.

SI. NO	Target Protein	ΔGbind (kcal/mol)
1	ExoU	−57.36
2	ExoS	−54.89
3	ExoT	−56.98
4	ExoY	−50.72
5	PLY	−59.18
6	SPI1	−49.21

**Table 3:** Quantum chemical descriptors of aloeresin-A.

SI. No	Descriptors	Definition	Value (eV)
1	E_HOMO_	-	−6.07
2	E_LUMO_	-	−1.70
3	Energy gap (ΔE)	∆E = E_LUMO_ − E_HOMO_	4.37
4	Electron affinity (A)	−E_LUMO_	1.70
5	Ionization potential (I)	−E_HOMO_	6.07
6	Electron accepting capacity (ω^+^)	(I + 3A)^2^/16(I − A)	1.78
7	Electron donating capacity (ω^−^)	(3I + A)^2^/16(I − A)	5.66
8	Chemical hardness (η)	(I-A)/2	2.18
9	Global softness (S)	1/2η	0.22
10	Chemical softness (σ)	1/η	0.45
11	Electrophilicity index (ω)	μ^2^/2η	3.45
12	Nucleophilicity index (N)	E_HOMO_-E_HOMO(TCE)_	3.11
13	Electronegativity (χ)	(I + A)/2	3.88
14	Electronic chemical potential (μ)	−(I + A)/2	−3.88

## Data Availability

The data used to support the findings of this study are included within the article.

## Supplementary Materials

The following supporting information can be downloaded at: https://www.mdpi.com/article/10.3390/molecules28196955/s1, Figure S1: Ligand RMSF plot for the aloeresin-A with ExoU (A), ExoS (B), ExoT (C), ExoY (D), PLY (E), and SPI1 (F). Figure S2: Interaction fraction plots of aloeresin-A with ExoU (A), ExoS (B), ExoT (C), ExoY (D), PLY (E), and SPI1 (F). Figure S3: Timeline of interactions between aloeresin-A with ExoU (A), ExoS (B), ExoT (C), ExoY (D), PLY (E), and SPI1 (F). Figure S4: Quantum chemical calculations of aloeresin-A. Table S1: Top-100 up-regulated genes. Table S2: Calculated structural parameters of aloeresin-A. Table S3: Mulliken charge distribution of aloeresin-A.
