# Supplementary material for: Multi-Target Effect of Aloeresin-A against Bacterial and Host Inflammatory Targets Benefits Contact Lens-Related Keratitis: A Multi-Omics and Quantum Chemical Investigation
Source: Molecules. 2023 Oct 6;28(19):6955. doi: 10.3390/molecules28196955 (PMC10574460; doi:10.3390/molecules28196955)

Table S1. Top-100 upregulated genes obtained by DGE analysis between bacteria infected and healthy corneal tissues.

SI.No	Genes	SI.No	Genes
1	*MMP9*	51	*LAPTM5*
2	*CXCL5*	52	*CXCR4*
3	*PI3*	53	*GPR84*
4	*AQP9*	54	*CCL20*
5	*IL1B*	55	*PLA2G7*
6	*C15orf48*	56	*GK*
7	*MCEMP1*	57	*PPBP*
8	*PLEK*	58	*MMP12*
9	*CLEC4A*	59	*TREM1*
10	*CD53*	60	*FCGR2A*
11	*FCN1*	61	*MS4A7*
12	*LILRB3*	62	*LTB*
13	*MARCO*	63	*CD48*
14	*OSM*	64	*SLAMF8*
15	*FCER1G*	65	*CYTH4*
16	*SOD2*	66	*ITGAX*
17	*CXCL6*	67	*FPR3*
18	*CSF3R*	68	*HLA-DRB5*
19	*RAC2*	69	*SPI1*
20	*CXCL8*	70	*NCF2*
21	*SLC11A1*	71	*GBP5*
22	*SPP1*	72	*S100A9*
23	*VCAN*	73	*CYBB*
24	*SRGN*	74	*MMP10*
25	*FPR2*	75	*HCK*
26	*SAMSN1*	76	*FERMT3*

27	*LYZ*	77	*IL1A*
28	*KRT6B*	78	*FCGR2B*
29	*CCL3L1*	79	*CCR1*
30	*PROK2*	80	*CD300A*
31	*CCL3L3*	81	*CHI3L2*
32	*FGR*	82	*KYNU*
33	*IL7R*	83	*CD69*
34	*SLA*	84	*ALOX5AP*
35	*TYROBP*	85	*IL6*
36	*NAMPT*	86	*PLAUR*
37	*SERPINA1*	87	*PILRA*
38	*SIGLEC14*	88	*CCRL2*
39	*LILRA3*	89	*VNN2*
40	*CCL3*	90	*MMP1*
41	*ITGAM*	91	*ADORA2A*
42	*CD14*	92	*NLRP3*
43	*IFI30*	93	*LYN*
44	*LCP1*	94	*C3AR1*
45	*FPR1*	95	*TNFRSF1B*
46	*CCL7*	96	*ITGB2*
47	*DEFB4A*	97	*HLA-DRB1*
48	*TNFAIP6*	98	*G0S2*
49	*CCL4L1*	99	*BCAT1*
50	*ADAMDEC1*	100	*WAS*

Table S2. Calculated geometrical parameters like bond length, bond angle and dihedral angles of aloeresin-A

Atoms	Bond length (Å)	Atoms	Bond angle (°)	Atoms	Dihedral angle (°)
O1-C14	1.44	C16-O1-C14	116.88	O1-C14 -C12 -O2	-73.87
O2-C12	1.43	H47-O3-C13	108.55	O1-C14 -C12 -C13	46.91
O3-C13	1.42	C26-O5-C19	119.90	O1-C14 -C12 -H40	171.14
O4-C15	1.42	H55-O7-C20	113.01	O1-C14 -C17 -C19	45.42
O5-C19	1.39	C13-C12-O2	107.48	O1-C14 -C17 -C20	-136.69
O6-C18	1.41	C14-C12-C13	113.43	O1-C16 -C15 -O4	174.48
O7-C20	1.37	H40-C12-C13	109.18	O1-C16 -C15 -C13	-58.72
O8-C24	1.23	C12-C13-O3	112.20	O1-C16 -C15 -H43	58.98
O10-C31	1.23	C15-C13-C12	111.33	O1-C16 -C18 -O6	167.23
O11-H67	0.96	H41-C13-C12	109.0	O1-C16 -C18 -H45	49.56
C12-C14	1.57	C12-C14-O1	108.36	O1-C16 -C18 -H46	-69.80
C13-C15	1.55	C17-C14-C12	121.70	O2-C12 -C13 -O3	-156.61
C14-C17	1.56	H42-C14-C12	107.34	O2-C12 -C13 -C15	76.70
C15-C16	1.55	C13-C15-O4	111.82	O2-C12 -C13 -H41	-43.02
C16-C18	1.54	C16-C15-C13	110.31	O2-C12 -C14 -C17	155.02
C17-C19	1.41	H43-C15-C13	108.03	O2-C12 -C14 -H42	39.50
C18-H45	1.09	C15-C16-O1	107.20	O2-C24 -C30 -C32	-178.2
C19-C21	1.42	C18-C16-C15	114.97	O2-C24 -C30 -H58	1.50
C21-C22	1.41	H44-C16-C15	110.77	O3-C13 -C12 -C14	80.87
C22-C23	1.39	C19-C17-C14	117.71	O3-C13 -C12 -H40	-44.91
C23-H49	1.08	C20-C17-C19	117.23	O3-C13 -C15 -O4	52.54
C25-C28	1.46	H45-C18-O6	106.21	O3-C13 -C15 -C16	-75.47
C26-C29	1.52	H46-C18-O6	109.50	O3-C13 -C15 -H43	167.01
C27-H52	1.09	H46-C18-H45	107.75	O4-C15 -C13 -C12	179.22
C28-H54	1.08	C21-C19-O5	121.14	O4-C15 -C13 -H41	-60.78
C29-H56	1.09	C17-C20-O7	123.68	O4-C15 -C16 -C18	55.22
C30-C32	1.35	C23-C20-C17	120.53	O4-C15 -C16 -H44	-67.57
C31-C34	1.52	C25-C21-C19	119.03	O5-C19 -C17 -C14	4.45
C32-H59	1.08	C23-C22-C21	119.65	O5-C19 -C17 -C20	-173.59
C33-C36	1.40	C27-C22-C23	116.84	O5-C19 -C21 -C22	176.81
C34-H61	1.09	H49-C23-C20	118.84	O5-C19 -C21 -C25	-2.82
C35-C37	1.39	O8-C24 -O2	124.50	O5-C26 -C28 -C25	0.44
C36-C38	1.39	C30-C24-O8	123.81	O5-C26 -C28 -H54	-179.08
C37-C39	1.39	C28-C25-O9	121.81	O5-C26 -C29 -C31	-71.0
C38-C39	1.39	C28-C26-O5	123.94	O5-C26 -C29 -H56	50.83
O1-C16	1.43	C29-C26-C28	125.49	O5-C26 -C29 -H57	168.08
O2-C24	1.36	H52-C27-C22	110.59	O6-C18 -C16 -C15	-73.59
O3-H47	0.96	H53-C27-C22	110.63	O6-C18 -C16 -H44	50.66
O4 -H48	0.96	H53-C27-H52	108.90	O7-C20 -C17 -C14	-4.43
O5-C26	1.36	H54-C28-C25	119.55	O7-C20 -C17 -C19	173.46
O6-H50	0.96	C31-C29-C26	111.85	O7-C20 -C23 -C22	-176.67
O7-H55	0.96	H56-C29-C31	109.88	O7-C20 -C23 -H49	2.66
O9-C25	1.23	H57-C29-C31	108.80	O8-C24 -O2-C12	-1.34
O11-C39	1.37	C32-C30-C24	122.73	O8-C24 -C30 -C32	1.52
C12-C13	1.55	H58-C30-C32	120.93	O8-C24 -C30 -H58	-178.76

C12 -H40	1.09	C34-C31-O10	121.16	O9-C25 -C21 -C19	-177.27
C13-H41	1.09	C33-C32-C30	126.07	O9-C25 -C21 -C22	3.10
C14-H42	1.09	H59-C32-C33	115.03	O9-C25 -C28 -C26	178.34
C15-H43	1.09	C36-C33-C32	122.16	O9-C25 -C28 -H54	-2.13
C16-H44	1.09	H60-C34-C31	110.41	O10 -C31 -C29 -C26	-63.22
C17-C20	1.41	H61-C34-H60	108.34	O10 -C31 -C29 -H56	175.33
C18-H46	1.09	H62-C34-H60	108.67	O10 -C31 -C29 -H57	58.27
C20-C23	1.39	C37-C35-C33	120.43	O10 -C31 -C34 -H60	123.31
C21-C25	1.52	H63-C35-C37	119.07	O10 -C31 -C34 -H61	3.36
C22-C27	1.53	H64-C36-C33	121.04	O10 -C31 -C34 -H62	-116.57
C24-C30	1.46	C39-C37-C35	119.74	O11 -C39 -C37 -C35	-179.93
C26-C28	1.34	H65-C37-C39	120.40	O11 -C39 -C37 -H65	0.000254
C27-H51	1.09	H66-C38-C36	119.78	O11 -C39 -C38 -C36	179.89
C27-H53	1.09	C37-C39-O11	119.76	O11 -C39 -C38 -H66	-0.11
C29-C31	1.53	C38-C39-C37	120.44	C12 -O2-C24 -C30	178.38
C29-H57	1.09	C24-O2-C12	122.51	C12 -C13 -O3-H47	-57.85
C30-H58	1.08	H48-O4-C15	108.51	C12 -C13 -C15 -C16	51.20
C32-C33	1.48	H50-O6-C18	108.55	C12 -C13 -C15 -H43	-66.30
C33-C35	1.40	H67-O11-C39	112.88	C12 -C14 -O1-C16	-59.75
C34-H60	1.09	C14-C12-O2	110.54	C12 -C14 -C17 -C19	175.22
C34-H62	1.09	H40-C12-O2	103.56	C12 -C14 -C17 -C20	-6.89
C35-H63	1.08	H40-C12-C14	112.10	C13 -C12 -O2-C24	156.83
C36-H64	1.08	C15-C13-O3	112.19	C13 -C12 -C14 -C17	-84.18
C37-H65	1.08	H41-C13-O3	103.14	C13 -C12 -C14 -H42	160.29
C38-H66	1.08	H41-C13-C15	108.56	C13 -C15 -O4-H48	-62.49
		C17-C14-O1	111.43	C13 -C15 -C16 -C18	-177.97
		H42-C14-O1	105.40	C13 -C15 -C16 -H44	59.22
		H42-C14-C17	101.15	C14 -O1-C16 -C15	66.41
		C16-C15-O4	114.03	C14 -O1-C16 -C18	-169.54
		H43-C15-O4	104.51	C14 -O1-C16 -H44	-53.14
		H43-C15-C16	107.72	C14 -C12 -O2-C24	-78.88
		C18-C16-O1	107.36	C14 -C12 -C13 -C15	-45.80
		H44-C16-O1	108.29	C14 -C12 -C13 -H41	-165.52
		H44-C16-C18	108.03	C14 -C17 -C19 -C21	-177.15
		C20-C17-C14	125.03	C14 -C17 -C20 -C23	177.22
		C16-C18-O6	111.84	C15 -C13 -O3-H47	68.35
		H45-C18-C16	109.86	C15 -C13 -C12 -H40	-171.59
		H46-C18-C16	111.47	C15 -C16 -C18 -H45	168.72
		C17-C19-O5	116.0	C15 -C16 -C18 -H46	49.36
		C21-C19-C17	122.85	C16 -O1-C14 -C17	76.70
		C23-C20-O7	115.77	C16 -O1-C14 -H42	-174.41
		C22-C21-C19	117.75	C16 -C15 -O4-H48	63.51
		C25-C21-C22	123.21	C16 -C15 -C13 -H41	171.19
		C27-C22-C21	123.50	C16 -C18 -O6-H50	-178.55
		C22-C23-C20	121.80	C17 -C14 -C12 -H40	40.04
		H49-C23-C22	119.36	C17 -C19 -O5-C26	178.77
		C30-C24-O2	111.69	C17 -C19 -C21 -C22	-1.49
		C21-C25-O9	124.22	C17 -C19 -C21 -C25	178.87
		C28-C25-C21	113.97	C17 -C20 -O7-H55	-178.55

		C29-C26-O5	110.51	C17 -C20 -C23 -C22	1.80
		H51-C27-C22	112.36	C17 -C20 -C23 -H49	-178.86
		H52-C27-H51	107.16	C18 -C16 -C15 -H43	-60.26
		H53-C27-H51	107.03	C19 -O5-C26 -C28	0.93
		C26-C28-C25	121.91	C19 -O5-C26 -C29	-176.47
		H54-C28-C26	118.54	C19 -C17 -C14 -H42	-66.17
		H56-C29-C26	109.19	C19 -C17 -C20 -C23	-4.88
		H57-C29-C26	109.83	C19 -C21 -C22 -C23	-1.80
		H57-C29-H56	107.18	C19 -C21 -C22 -C27	179.57
		H58-C30-C24	116.34	C19 -C21 -C25 -C28	3.85
		C29-C31-O10	121.54	C20 -C17 -C14 -H42	111.7
		C34-C31-C29	117.29	C20 -C17 -C19 -C21	4.79
		H59-C32-C30	118.89	C20 -C23 -C22 -C21	1.67
		C35-C33-C32	118.53	C20 -C23 -C22 -C27	-179.61
		C36-C33-C35	119.31	C21 -C19 -O5-C26	0.35
		H61-C34-C31	110.67	C21 -C22 -C23 -H49	-177.65
		H62-C34-C31	110.29	C21 -C22 -C27 -H51	-179.68
		H62-C34-H61	108.40	C21 -C22 -C27 -H52	60.61
		H63-C35-C33	120.50	C21 -C22 -C27 -H53	-60.12
		C38-C36-C33	120.32	C21 -C25 -C28 -C26	-2.75
		H64-C36-C38	118.64	C21 -C25 -C28 -H54	176.76
		H65-C37-C35	119.86	C22 -C21 -C25 -C28	-175.75
		C39-C38-C36	119.76	C23 -C20 -O7-H55	-0.13
		H66-C38-C39	120.46	C23 -C22 -C21 -C25	177.81
		C38-C39-O11	119.80	C23 -C22 -C27 -H51	1.64
				C23 -C22 -C27 -H52	-118.04
				C23 -C22 -C27 -H53	121.21
				C24 -O2-C12 -H40	41.35
				C24 -C30 -C32 -C33	-179.57
				C24 -C30 -C32 -H59	-0.53
				C25 -C21 -C22 -C27	-0.80
				C25 -C28 -C26 -C29	177.46
				C26 -C29 -C31 -C34	117.35
				C27 -C22 -C23 -H49	1.05
				C28 -C26 -C29 -C31	111.63
				C28 -C26 -C29 -H56	-126.52
				C28 -C26 -C29 -H57	-9.27
				C29 -C26 -C28 -H54	-2.06
				C29 -C31 -C34 -H60	-57.26
				C29 -C31 -C34 -H61	-177.2
				C29 -C31 -C34 -H62	62.85
				C30 -C32 -C33 -C35	-161.45
				C30 -C32 -C33 -C36	17.78
				C32 -C33 -C35 -C37	179.33
				C32 -C33 -C35 -H63	-0.47
				C32 -C33 -C36 -C38	-179.34
				C32 -C33 -C36 -H64	1.07
				C33 -C32 -C30 -H58	0.73
				C33 -C35 -C37 -C39	-0.03

				C33 -C35 -C37 -H65	-179.97
				C33 -C36 -C38 -C39	0.11
				C33 -C36 -C38 -H66	-179.87
				C34 -C31 -C29 -H56	-4.08
				C34 -C31 -C29 -H57	-121.14
				C35 -C33 -C32 -H59	19.47
				C35 -C33 -C36 -C38	-0.11
				C35 -C33 -C36 -H64	-179.68
				C35 -C37 -C39 -C38	0.03
				C36 -C33 -C32 -H59	-161.28
				C36 -C33 -C35 -C37	0.07
				C36 -C33 -C35 -H63	-179.73
				C36 -C38 -C39 -C37	-0.07
				C37 -C39 -O11 -H67	179.8
				C37 -C39 -C38 -H66	179.91
				C38 -C39 -O11 -H67	-0.16
				C38 -C39 -C37 -H65	179.96
				C39 -C37 -C35 -H63	179.78
				C39 -C38 -C36 -H64	179.69
				H40 -C12 -C13 -H41	68.67
				H40 -C12 -C14 -H42	-75.48
				H41 -C13 -O3-H47	-175.01
				H41 -C13 -C15 -H43	53.68
				H43 -C15 -O4-H48	-179.12
				H43 -C15 -C16 -H44	176.93
				H44 -C16 -C18 -H45	-67.01
				H44 -C16 -C18 -H46	173.62
				H45 -C18 -O6-H50	-58.71
				H46 -C18 -O6-H50	57.37
				H58 -C30 -C32 -H59	179.77
				H63 -C35 -C37 -H65	-0.14
				H64 -C36 -C38 -H66	-0.28

Table S3. Mulliken atomic charge distribution of aloeresin-A

Atom	Charge (a.u)	Atom	Charge (a.u)
O1	-0.51	C32	-0.05
O2	-0.52	C33	0.05
O3	-0.54	C34	-0.40
O4	-0.54	C35	-0.11
O5	-0.50	C36	-0.10
O6	-0.55	C37	-0.14
O7	-0.54	C38	-0.16
O8	-0.51	C39	0.35
O9	-0.52	H40	0.15
O10	-0.45	H41	0.12
O11	-0.54	H42	0.14
C12	0.15	H43	0.11
C13	0.13	H44	0.07
C14	0.13	H45	0.10
C15	0.17	H46	0.08
C16	0.14	H47	0.31
C17	-0.14	H48	0.30
C18	0.05	H49	0.09
C19	0.34	H50	0.31
C20	0.36	H51	0.09
C21	-0.09	H52	0.14
C22	0.07	H53	0.15
C23	-0.21	H54	0.11
C24	0.61	H55	0.33
C25	0.45	H56	0.13
C26	0.37	H57	0.15
C27	-0.33	H58	0.10
C28	-0.18	H59	0.12
C29	-0.31	H60	0.16
C30	-0.16	H61	0.13
C31	0.45	H62	0.13
		H63	0.11
		H64	0.11
		H65	0.12
		H66	0.09
		H67	0.32

Figure S1. RMSF plot of aloeresin-A fit on ExoU (A), ExoS (B), ExoT (C), ExoY (D), PLY (E) and SPI1 (F)

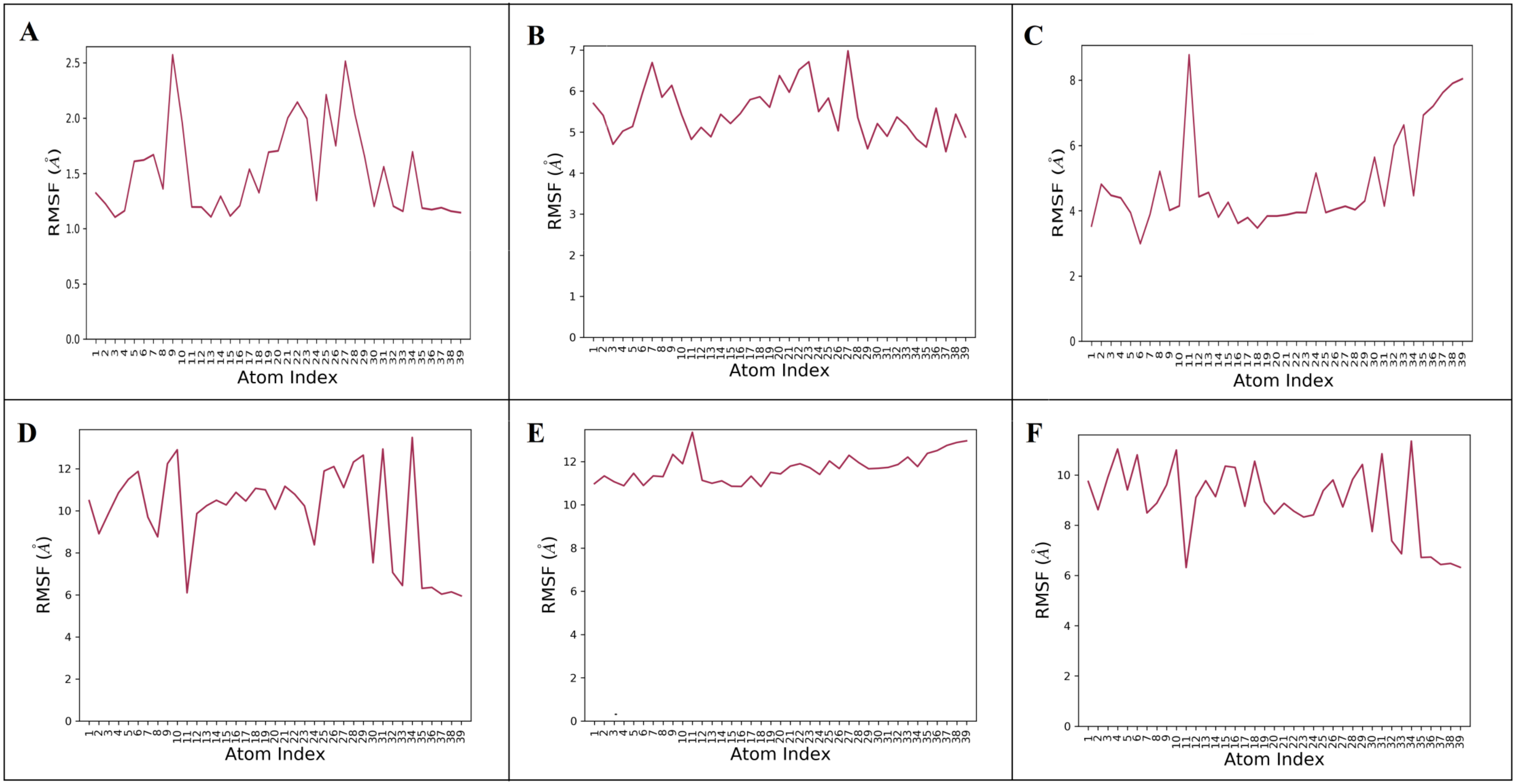

Figure S2. Interaction fraction plot of aloeresin-A with ExoU (A), ExoS (B), ExoT (C), ExoY (D), PLY (E) and SPI1 (F). Color codes- Green: hydrogen bonds; Violet: Hydrophobic; Pink: Ionic bonds; Blue: Water bridges

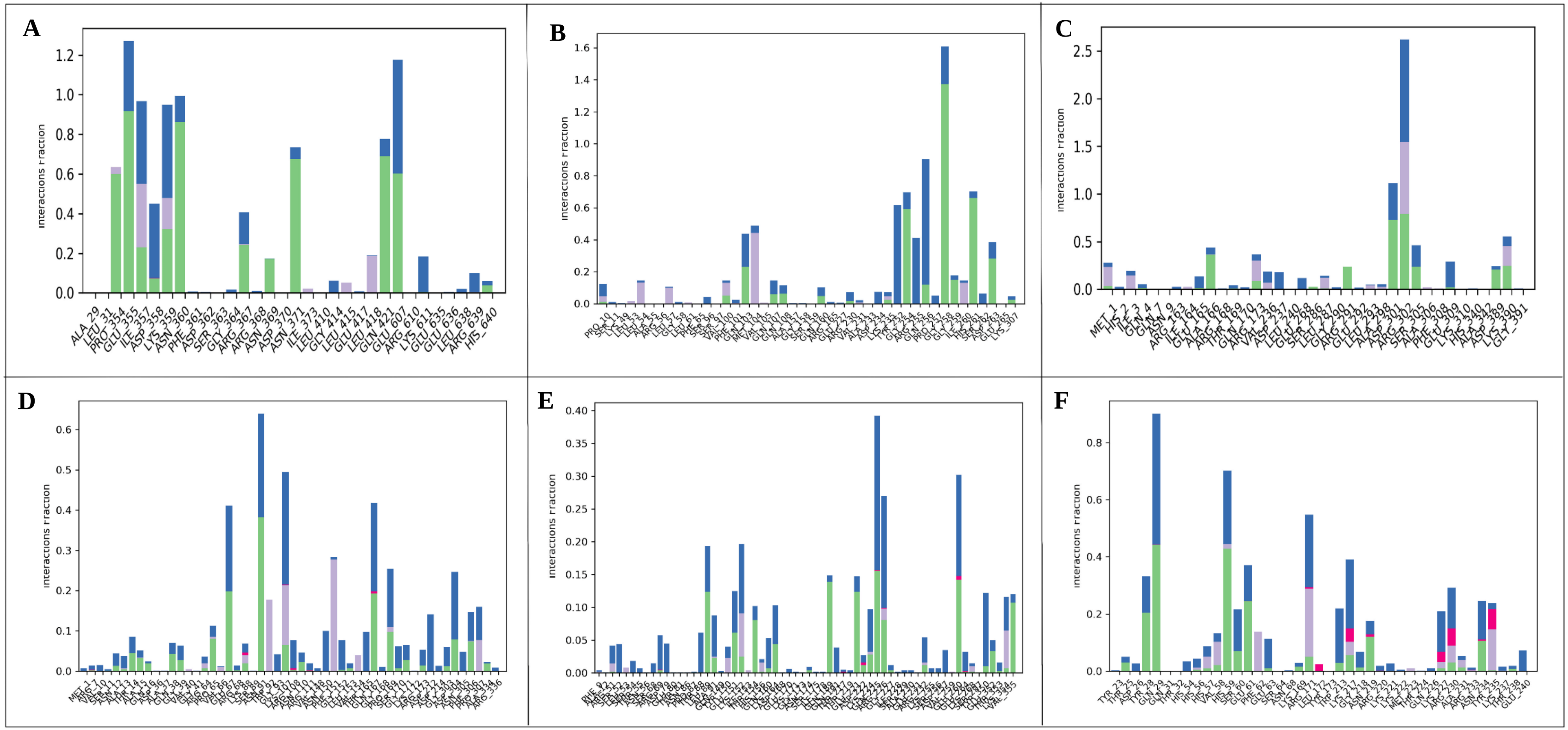

Figure S3. Timeline of interactions of aloeresin-A with ExoU (A), ExoS (B), ExoT (C), ExoY (D), PLY (E) and SPI1 (F)

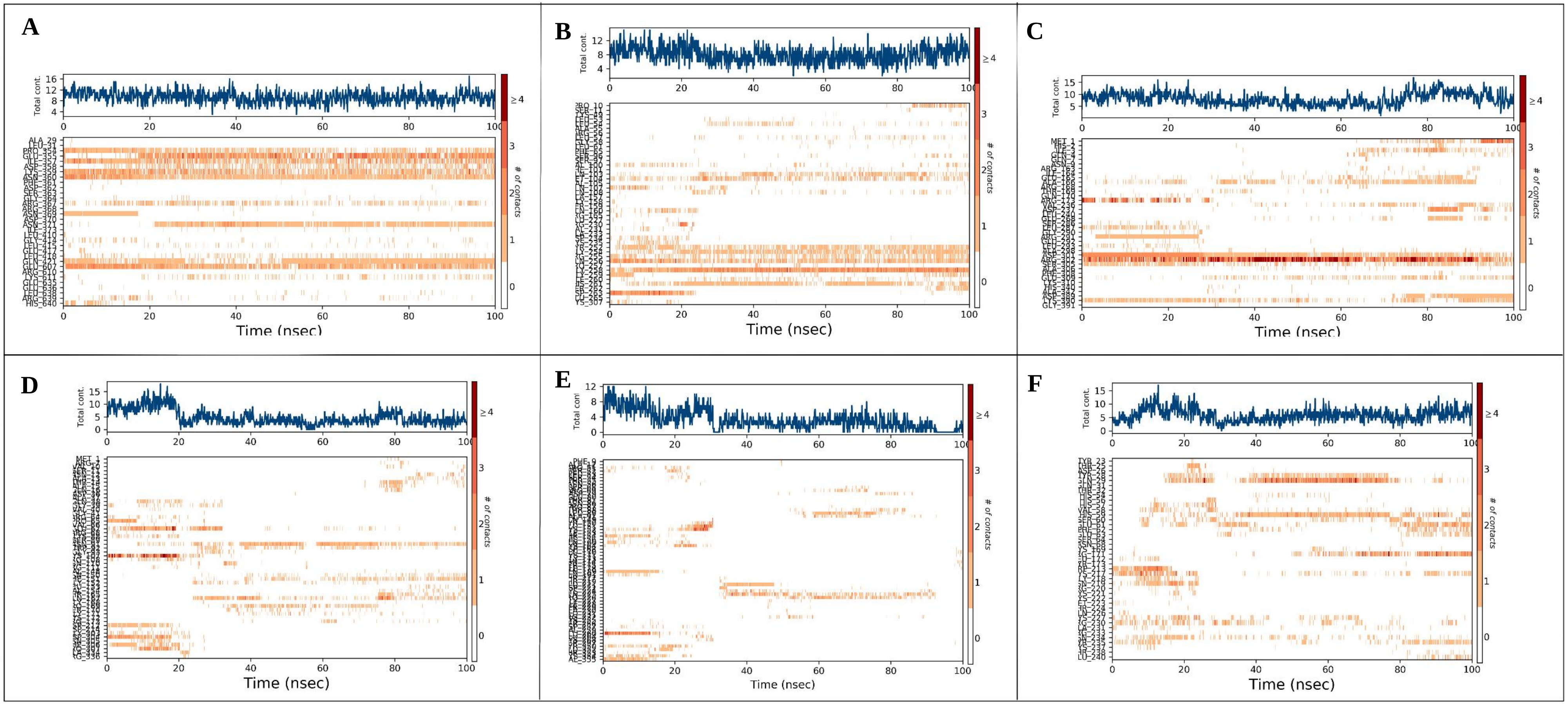

Figure S4. Density Functional theory calculations of aloeresin-A – Molecular structure (A), Energy gap (B), molecular electrostatic potential (C) and Mulliken charge distribution (D)

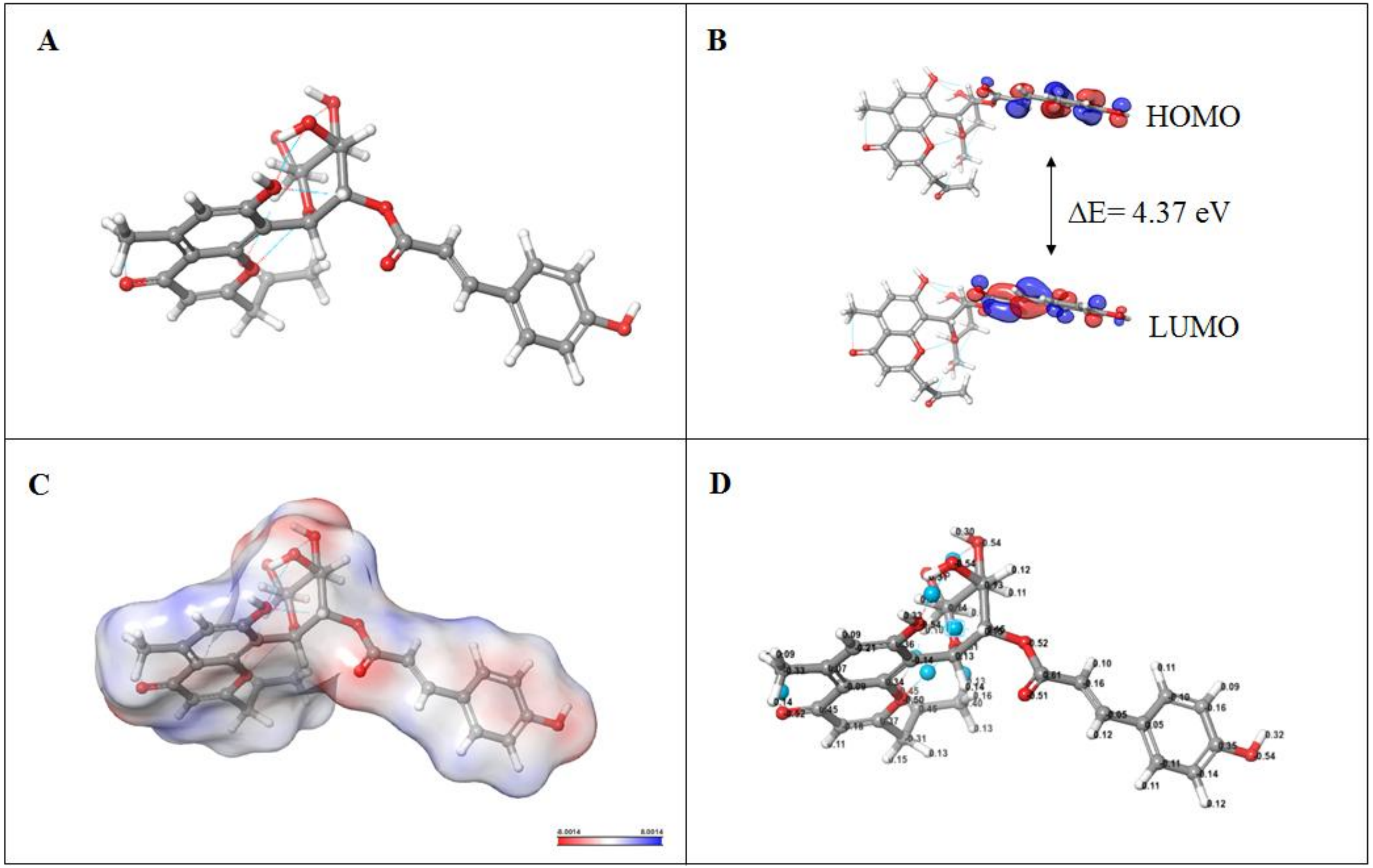

Supplement: Supplementary file 1 [file molecules-28-06955-s001.zip › molecules-2602792-supplementary.pdf]
